# Maximizing capture of gene co-expression relationships through pre-clustering of input expression samples: an *Arabidopsis* case study

**DOI:** 10.1186/1752-0509-7-44

**Published:** 2013-06-05

**Authors:** F Alex Feltus, Stephen P Ficklin, Scott M Gibson, Melissa C Smith

**Affiliations:** 1 Department of Genetics & Biochemistry, Clemson University, 105 Collings Street, Clemson, SC 29634, USA; 2 Plant and Environmental Sciences, Clemson University, 105 Collings Street, Clemson, SC 29634, USA; 3 The Holcombe Department of Electrical and Computer Engineering, Clemson, SC 29634, USA

**Keywords:** Gene network, Arabidopsis, Systems biology, Relevance network

## Abstract

**Background:**

In genomics, highly relevant gene interaction (co-expression) networks have been constructed by finding significant pair-wise correlations between genes in expression datasets. These networks are then mined to elucidate biological function at the polygenic level. In some cases networks may be constructed from input samples that measure gene expression under a variety of different conditions, such as for different genotypes, environments, disease states and tissues. When large sets of samples are obtained from public repositories it is often unmanageable to associate samples into condition-specific groups, and combining samples from various conditions has a negative effect on network size. A fixed significance threshold is often applied also limiting the size of the final network. Therefore, we propose pre-clustering of input expression samples to approximate condition-specific grouping of samples and individual network construction of each group as a means for dynamic significance thresholding. The net effect is increase sensitivity thus maximizing the total co-expression relationships in the final co-expression network compendium.

**Results:**

A total of 86 *Arabidopsis thaliana* co-expression networks were constructed after *k*-means partitioning of 7,105 publicly available ATH1 Affymetrix microarray samples. We term each pre-sorted network a Gene Interaction Layer (GIL). Random Matrix Theory (RMT), an un-supervised thresholding method, was used to threshold each of the 86 networks independently, effectively providing a dynamic (non-global) threshold for the network. The overall gene count across all GILs reached 19,588 genes (94.7% measured gene coverage) and 558,022 unique co-expression relationships. In comparison, network construction without pre-sorting of input samples yielded only 3,297 genes (15.9%) and 129,134 relationships. in the global network.

**Conclusions:**

Here we show that pre-clustering of microarray samples helps approximate condition-specific networks and allows for dynamic thresholding using un-supervised methods. Because RMT ensures only highly significant interactions are kept, the GIL compendium consists of 558,022 unique high quality *A. thaliana* co-expression relationships across almost all of the measurable genes on the ATH1 array. For *A. thaliana*, these networks represent the largest compendium to date of significant gene co-expression relationships, and are a means to explore complex pathway, polygenic, and pleiotropic relationships for this focal model plant. The networks can be explored at sysbio.genome.clemson.edu. Finally, this method is applicable to any large expression profile collection for any organism and is best suited where a knowledge-independent network construction method is desired.

## Background

Cellular processes underlying expression of complex traits have a major impact on health, agriculture, and our understanding of general biology. The gene co-expression network (GCN) models coordinated gene expression across a series of input data sets such as microarrays [1,2]. In a GCN, nodes represent genes, and edges describe significant gene co-expression relationships. The GCN also exhibits properties common to most naturally occurring networks such as scale-free, small world and hierarchical topology [3,4]. Due to the availability of large quantities of publically available expression data and the relative ease of construction, GCNs have been constructed for a broad array of organisms including human [2,5,6], yeast [7-9], Arabidopsis [10-13], rice [14,15], maize [16], potato [17] and many more. These networks have elucidated gene sets involved in varied biological systems including cell wall biosynthesis [13], mouse weight [18], and complex trait expression [19-22].

A GCN is constructed by performing pair-wise correlation analysis of every gene on the array. Typically, Pearson’s Correlation Coefficient (PCC) is used, and a large *n* x *n* matrix of PCC values is obtained where *n* is the number of measurable genes. An analytical method is then employed to identify the level at which correlation values should be thresholded to yield biologically meaningful co-expression relationships. Often, co-expression networks are built for analysis of differentially connected genes between two or more experimental conditions (environment, disease state, genotype, or tissue type) [23-25]. In these cases networks are constructed separately for each condition and the context of the connectivity can then be examined to identify modules (or gene sets) putatively causal for phenotypic traits. Genes with known causality can be used to guide selection of modules.

In some cases, global co-expression networks are created using a large number of samples from publicly available repositories—the goal being to mine knowledge across the compendium of samples not previously identified through individual experiments. For thousands or tens-of-thousands of samples two challenges occur. First, classification of samples into conditions becomes manually intractable and sample descriptions are often insufficient to automate proper classification. Therefore, conditional context is often ignored, samples are combined and similarity measurements are calculated across all samples. However, this treats all genes and samples (with varying conditions) equally when calculating similarity scores. As the number of samples with different conditions increases in the input dataset, the number of significant co-expression relationships decreases [26]. Therefore, networks built from a set of samples with a large number of conditions tend to be small. Cheng and Church recognized the illogic of weighting all genes and samples the same for similarity calculation and proposed biclustering of expression data [27] to generate biclusters of genes with similar expression in similar contexts (conditions). Many types of biclustering methods have been developed [28]. However, for large disparate sample sets, the difficulty in classifying samples into conditional groups makes biclustering difficult.

A second challenge for network construction is identification of a proper significance threshold. Many methods have been employed for significance thresholding. These include *ad hoc* methods [1,29-31], permutation testing [5], linear regression [13], rank-based methods [32,33], Fisher’s test of homogeneity [34], spectral graph theory [35], Random Matrix Theory (RMT) [36,37], Partial Correlation and Information Theory (PCIT) [26], methods that use topological properties [38], and supervised machine learning [39,40]. In some cases a constant threshold is applied to the entire network. While significant relationships can be found using a constant threshold, a dynamic threshold is better suited for context-dependent co-expression variability. Dynamic thresholding can increase sensitivity and decrease false-positives. Rank-based methods, PCIT and supervised machine learning methods all employ a form of dynamic thresholding.

**Figure 1:**
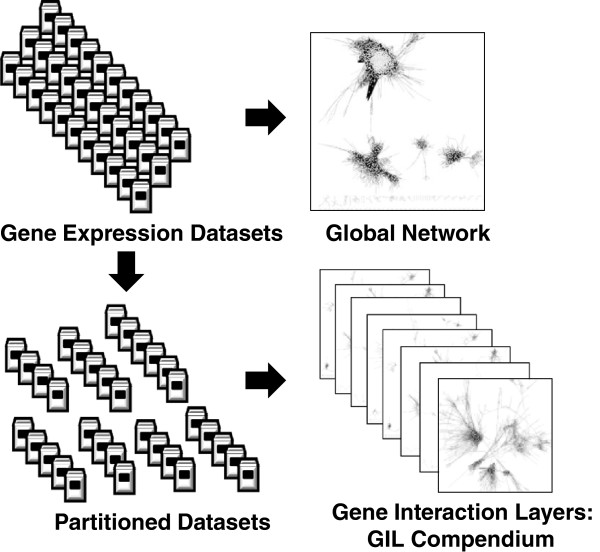
**Deconstructing a global network into gene interaction layers (GILs).** Gene expression datasets can be used to construct a co-expression network in total (global) or sorted by expression pattern into groups prior to network construction (GILs).

To address the challenges of large sample, large conditional network construction, we use a method of segregating input samples into groups before network construction. Without knowledge of sample conditions, we approximate expression conditions by pre-clustering of input gene-expression profiles into groups, and apply dynamic significance thresholding through independent network construction for each sample group. We refer to the network from each group as a Gene Interaction Layer (GIL). The GIL compendium represents an attempt to maximize the capture of all possible interactions across all samples (Figure 1). The GIL compendium also allows for gene and gene-gene interactions to exist in multiple GILs and therefore provides a framework for the analysis of intersecting biological pathways and potentially pleiotropic interactions. To demonstrate the effectiveness of this approach, we generated a GIL collection for the model plant *Arabidopsis thaliana* (Arabidopsis) by pre-clustering 7,105 input samples. Our results indicate that the Arabidopsis GIL compendium represents a dramatic improvement in capture of gene co-expression relationships.

## Results

Arabidopsis GILs were constructed by partitioning 7,105 publicly available Affymetrix ATH1 microarray RNA expression samples using a network construction pipeline [41] which implements Random Matrix Theory (RMT) for biological signal thresholding [9]. Samples were RMA [42] normalized prior to pre-clustering. Additionally, a typical “global” network was constructed using all normalized input samples. The global network comprised 3,297 nodes and 129,134 edges (average degree, <*k*> = 78) representing 15.9% of the known *Arabidopsis* genes as measured by the ATH1 platform. As mentioned previously, this small network size is expected given the mixture of multiple sample conditions. To improve capture of measurable genes and co-expression relationships, we partitioned the input samples into *K* groups with similar expression patterns using the K-means clustering algorithm [43]. Each cluster of samples were then RMA normalized again within their respective groups and co-expression networks were built for each cluster.

### The effects of *K* size on the GIL collection

**Figure 2:**
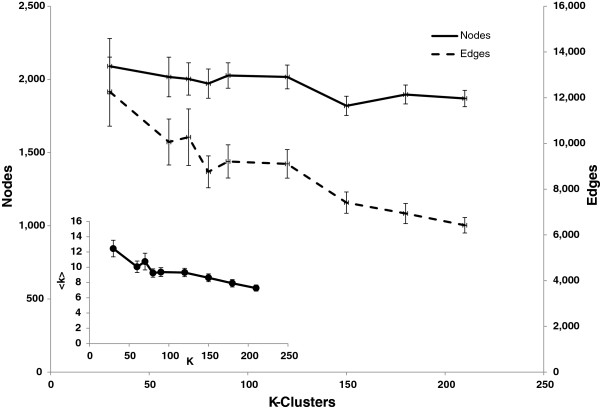
**Basic topological metrics of GILs constructed from varied partitioning sizes.** Primary graph shows average edge (dashed line) and average node (solid line) counts for GILs constructed from K-means of cluster size *K*. Inset graph shows average connectivity (<*k*>, black circles) for GILs constructed from K-means of cluster size *K*.

The choice of *K* size in k-means clustering should have an impact on the topological properties of each GIL. To quantify these differences we performed K-means clustering nine times at *K* sizes of 30, 60, 70, 80, 90, 120, 150, 180, and 210, yielding 990 total groups. The average array count for each *K* size ranged from 236.8 (*K*=30) to 33.8 (*K*=210) arrays. Changes in the various topological properties including clustering co-efficient, closeness, betweenness, page rank, etc., between different *K* sizes can be found in Additional file 1: Figure S1. In summary, relative to the global network, the average number of nodes in each group was lower and ranged from 2,090 (*K*=30) to 1,819 (*K*=150) (Figure 2). The average number of edges in each group was much lower relative to the global network and declined with increasing *K* size ranging from 12,265 (*K*=30) to 6,421 (*K*=210) (Figure 2).

**Figure 3:**
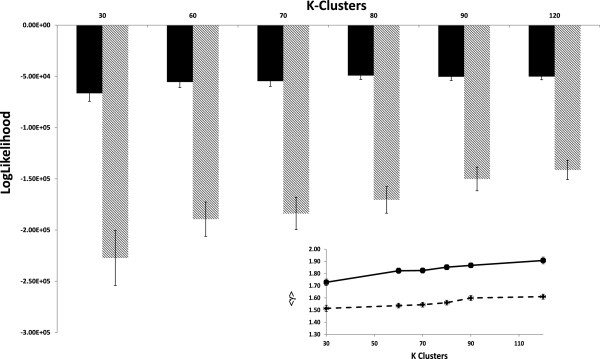
**GILs are scale free at varied partition sizes.** Primary graph shows log-likelihood fit to a Kronecker scale-free graph model at k-means cluster sizes *K*. Clusters were randomized at 0% (solid bars) and 50% (striped bars) prior to network construction. Inset graph shows average scaling exponent (<γ>) for GILs constructed from K-means of cluster size *K* at 0% (solid line) and 50% (dashed line) randomization.

To test if the degree of pre-clustering of samples had a negative effect on network topology, we randomly re-assigned 50% of the samples among the groups and reconstructed the networks. This re-assignment occurred at *K* sizes of 30, 50, 60, 70, 80, 90, and 120. Sample re-assignment had a noticeable effect on scale-free behavior as exhibited by a decrease fit to the Kronecker scale-free graph model [44] and decrease in average scaling exponent γ (Figure 3). The scaling exponent for the global network was 1.36. Therefore, the randomization of samples between pre-clustered groups seemed to generate networks with properties more similar to the “global” network. However, despite differences in fit to the Kronecker scale-free graph model and to the scaling exponent, all networks exhibited scale-free behavior; therefore each *K* size generated networks that appear realistic.

### Identification of the best *K* size

**Figure 4:**
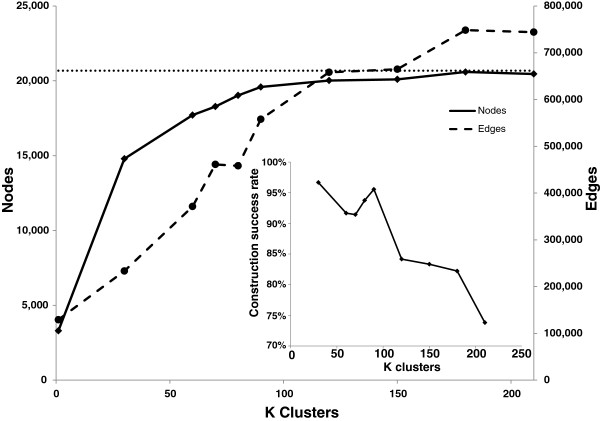
**Co-expression interaction capture.** Primary graph shows total unique node (solid line) and edge (dashed line) accumulation in all GIL collections at various cluster sizes, *K*. Dotted line at 23,244 nodes is the maximum number of genes measured by ATH1 array. Inset graph shows the success rate of possible GILs constructed from cluster size, *K*.

To identify the best *K* size, we examined the network from each *K* group for total gene capture, functional annotation enrichment, and module count. First, we examined total node capture among the GILs from each *K* group. While the global network captured 15.9% of possible genes measured by the array platform, the total gene space captured across all networks of a *K* group increased with increasing *K* size (Figure 4). At *K*=180, for example, 99.6% of all measurable genes were captured at least once. Second, we modularized the networks using MCL [45] and tested for function term enrichment (Bonferroni *p* < 0.001) for each module relative to whole genome background using Gene Ontology (GO) terms [46], KEGG pathways [47], Interpro protein domains [48], Plant Ontology (PO) terms [49], and AraCyc pathways [50]. In general, the number of total enriched terms tended to increase along with *K* size (Additional file 1: Figure S2). However, the number of unique enriched terms tended to plateau at *K*=90. Third, we investigated modular behavior by examining the average number of modules for each *K*. For this test we used a second module detection algorithm called link-community detection [51]. The link-community method allows for nodes to be present in more than one module which is more representative of shared genes found in intersecting pathways. The average number of link-community modules (LCMs) per GIL decreased as *K* size increased while the number of MCL modules rose slightly (Additional file 1: Figure S3). Therefore, to balance a maximum number of nodes, edges, functional enrichment, and high module count representing both coverage (MCL) and multi-functionality (LCM), *K*=90 was chosen as the K-means clustering size for the *Arabidopsis* GIL collection*.*

### Summary of the K 90 GIL collection

In summary, the K90 GIL collection consisted of 86 GILs and contained a total of 19,588 genes (94.7% measured gene coverage) and 558,022 unique co-expression relationships. Counting the experiments contained in the K90 GIL collection revealed that each GIL was comprised of multiple NCBI GEO experiments (μ = 78.9 arrays; σ=53.7; Additional file 2: Table S1) derived from a blend of GEO series (μ=12.3; σ=10.7), indicating that GILs were not simply the product of segregating datasets into the original experiment groups. After module detection using the Markov Clustering (MCL; [45]) and link-community [51] methods, we circumscribed 38,234 MCL modules, encapsulating 94.7% of measurable genes and 26,570 link-community modules capturing 71.0% of genes, each with a median occurrence of six genes per module (min = 2 and max = 707) (Table 1). Four GILs (43, 46, 52, 70) in the *K*90 collection did not construct due to high levels of correlation between genes. For MCL modules, we identified 867 enriched function terms (657 unique) within the global network while the K90 GIL collection yielded 37,614 enriched function terms (4,789 unique) — a 7.2-fold improvement in representation of unique functional terms in the GILs.

**Table 1:** K90 GIL Collection Overview

**GIL**^**A**^	**Arrays**	**Datasets**	**Nodes**	**Edges**	**<k>**	**RMT**	**γ**	**Modules**^**B**^	**GO**^**c**^	**Interpro**	**KEGG**^**C**^	**AraCyc**^**c**^	**PO**^**C**^	**GIL**^**A**^	**Arrays**	**Datasets**	**Nodes**	**Edges**	**<k>**	**RMT**	**γ**	**Modules**^**B**^	**GO**^**c**^	**Interpro**	**KEGG**^**C**^	**AraCyc**^**c**^	**PO**^**C**^
1	48	7	2,563	7,051	5.50	0.929	1.99	616/294	153/225	192/112	192/112	19/18	21/13	46	116	28	n.a.	n.a.	n.a.	n.a.	n.a.	n.a./n.a.	n.a./n.a.	n.a./n.a.	n.a./n.a.	n.a./n.a.	n.a./n.a.
2	38	2	1,921	7,807	8.13	0.943	1.85	418/226	98/135	84/50	84/50	14/19	11/34	47	105	12	1,445	4,969	6.88	0.910	1.85	280/282	122/123	119/110	54/90	15/15	10/19
3	76	22	2,919	6,415	4.40	0.844	2.13	763/251	313/219	298/130	298/130	28/20	45/36	48	269	31	2,638	11,228	8.51	0.803	1.78	489/493	316/663	231/317	103/200	35/38	45/62
4	72	12	2,764	6,565	4.75	0.872	2.07	713/319	334/448	303/168	303/168	38/17	45/23	49	39	2	1,954	9,688	9.92	0.935	1.76	413/376	177/286	160/133	78/86	15/10	14/3
5	40	2	997	4,346	8.72	0.936	1.86	197/136	115/107	81/35	81/35	11/7	3/2	50	113	27	3,520	11,996	6.82	0.809	1.92	770/497	330/350	284/222	144/99	30/5	21/14
6	123	25	2,446	15,547	12.71	0.835	1.75	507/458	254/854	203/360	203/360	24/19	18/24	51	45	9	2,377	4,570	3.85	0.911	2.20	651/223	153/132	173/75	90/43	16/9	4/3
7	75	22	2,491	4,259	3.42	0.842	2.26	705/229	267/148	251/77	251/77	21/13	10/8	52	8	1	n.a.	n.a.	n.a.	n.a.	n.a.	n.a./n.a.	n.a./n.a.	n.a./n.a.	n.a./n.a.	n.a./n.a.	n.a./n.a.
8	42	4	1,596	5,282	6.62	0.964	1.92	315/217	90/72	88/65	88/65	13/13	19/15	53	143	32	1,898	5,518	5.81	0.826	1.96	438/232	231/186	192/94	86/64	17/15	19/12
9	84	2	1,859	16,072	17.29	0.876	1.64	327/374	121/927	90/165	90/165	22/92	24/39	54	85	13	1,983	3,849	3.88	0.859	2.23	522/206	166/80	191/52	70/36	22/14	18/9
10	147	16	1,804	9,878	10.95	0.898	1.70	317/329	168/543	111/98	111/98	16/44	46/93	55	87	29	3,811	16,013	8.40	0.802	1.83	815/668	373/580	311/261	137/170	38/27	28/14
11	63	1	1,971	16,547	16.79	0.889	1.62	351/484	175/1070	148/402	148/402	19/39	15/46	56	40	4	1,414	13,540	19.15	0.965	1.54	206/448	87/540	63/87	23/103	8/3	46/265
12	124	14	1,257	5,985	9.52	0.875	1.75	212/294	63/93	47/43	47/43	5/16	12/8	57	27	3	1,944	7,628	7.85	0.916	1.91	506/229	106/319	125/65	52/81	13/17	32/36
13	49	5	1,293	3,808	5.89	0.952	1.96	295/158	59/84	79/39	79/39	7/16	1/2	58	131	17	1,861	8,262	8.88	0.865	1.81	367/360	283/694	183/200	79/186	38/84	33/73
14	87	10	3,286	37,336	22.72	0.867	1.58	583/779	218/1186	190/328	190/328	24/44	69/117	59	50	13	926	2,415	5.22	0.927	2.05	229/102	41/27	63/20	20/6	7/0	6/5
15	46	10	1,925	10,961	11.39	0.941	1.75	382/350	86/71	123/48	123/48	8/6	21/51	60	44	13	1,703	6,996	8.22	0.916	1.83	381/267	199/404	165/85	75/118	17/10	34/64
16	64	9	1,552	5,071	6.53	0.926	1.94	359/220	98/60	114/35	114/35	3/8	6/3	61	59	4	2,006	10,532	10.50	0.891	1.69	327/391	175/582	102/192	44/137	13/54	30/38
17	37	7	1,895	12,248	12.93	0.945	1.66	347/472	109/465	72/129	72/129	14/49	19/29	62	73	16	1,185	4,543	7.67	0.886	1.91	278/213	139/177	143/79	74/70	13/6	6/2
18	43	6	1,783	6,340	7.11	0.943	1.90	416/322	78/86	120/69	120/69	15/14	12/14	63	131	23	2,448	6,632	5.42	0.850	1.98	538/303	234/279	193/143	98/93	18/32	31/45
19	166	33	2,866	9,723	6.79	0.795	1.89	628/393	323/278	293/116	293/116	36/33	40/44	64	197	30	3,122	11,982	7.68	0.781	1.81	589/435	361/484	257/234	114/147	33/35	48/66
20	142	34	3,440	12,799	7.44	0.783	1.88	752/432	336/385	294/196	132/149	34/35	36/52	65	30	3	332	1,685	10.15	0.988	1.69	60/85	64/263	23/54	11/61	5/27	1/4
21	71	9	1,525	12,678	16.63	0.922	1.62	263/332	121/354	84/98	30/75	16/53	29/68	66	23	4	2,523	20,630	16.35	0.954	1.86	626/325	132/64	158/34	72/27	16/5	26/34
22	70	9	2,465	6,898	5.60	0.903	2.03	633/288	191/200	231/109	99/67	19/18	15/37	67	215	41	3,254	16,033	9.85	0.762	1.78	617/461	377/533	285/298	128/172	48/68	63/66
23	24	1	402	3,010	14.98	0.983	1.60	66/97	3/15	8/10	7/8	0/0	0/5	68	86	18	1,283	7,209	11.24	0.893	1.69	223/268	171/767	99/230	47/172	19/43	24/22
24	87	6	2,347	5,280	4.50	0.905	2.09	599/298	169/184	172/133	86/78	12/5	14/15	69	192	19	3,597	40,668	22.61	0.823	1.59	585/597	301/504	269/264	103/77	26/58	78/946
25	72	14	3,512	10,063	5.73	0.856	2.06	895/319	338/203	295/93	146/59	37/25	43/54	70	37	8	n.a.	n.a.	n.a.	n.a.	n.a.	n.a./n.a.	n.a./n.a.	n.a./n.a.	n.a./n.a.	n.a./n.a.	n.a./n.a.
26	32	5	1,792	26,789	29.90	0.920	1.62	413/280	41/23	72/32	33/9	11/11	28/244	71	235	24	2,034	7,791	7.66	0.859	1.85	410/282	231/250	202/141	78/70	16/33	39/137
27	72	1	634	2,043	6.44	0.966	1.91	128/103	52/52	57/46	24/24	4/3	6/3	72	24	2	1,314	4,931	7.51	0.948	1.88	279/262	47/39	68/28	22/25	7/18	13/6
28	75	9	593	5,428	18.31	0.961	1.57	96/128	21/21	28/20	12/24	5/16	1/3	73	114	16	2,145	13,061	12.18	0.836	1.76	454/403	131/126	176/131	70/66	18/43	21/41
29	129	22	2,798	9,343	6.68	0.814	1.91	595/366	299/282	254/153	118/107	37/26	23/12	74	42	7	1,244	3,508	5.64	0.956	2.00	284/183	55/26	92/16	46/34	8/13	4/0
30	104	9	2,107	21,381	20.30	0.870	1.60	363/495	128/314	115/168	66/88	18/73	19/28	75	161	44	2,689	13,042	9.70	0.791	1.84	554/342	247/579	262/247	112/158	34/15	12/12
31	40	10	2,715	7,370	5.43	0.895	2.06	725/293	147/156	217/144	100/103	32/27	23/54	76	34	4	1,198	8,819	14.72	0.930	1.66	215/313	46/104	65/190	22/14	9/24	7/32
32	104	9	1,918	6,878	7.17	0.910	1.86	420/387	247/488	154/106	82/137	16/39	13/13	77	70	9	2,340	5,730	4.90	0.893	2.07	591/291	239/315	194/103	110/77	20/19	10/5
33	61	4	1,409	5,306	7.53	0.923	1.94	349/200	81/35	111/36	44/13	12/5	7/4	78	172	31	3,513	15,437	8.79	0.777	1.76	665/570	451/880	361/392	150/287	53/45	40/107
34	34	4	2,213	7,181	6.49	0.932	1.92	524/238	111/109	153/74	56/29	16/13	52/56	79	26	6	2,599	5,542	4.26	0.927	2.22	741/225	124/67	186/44	85/25	12/5	14/13
35	49	9	2,004	6,306	6.29	0.935	1.93	458/297	217/394	155/125	88/107	19/41	9/16	80	112	30	3,288	23,432	14.25	0.821	1.75	628/621	294/324	246/134	124/75	31/29	23/19
36	18	1	1,365	7,141	10.46	0.952	1.79	2889/198	79/98	68/60	30/38	13/10	15/18	81	18	1	678	2,830	8.35	0.959	1.83	146/132	11/2	34/17	6/8	1/6	0/3
37	55	6	2,450	7,970	6.51	0.941	1.94	556/318	138/269	166/84	75/72	21/38	9/4	82	125	22	1,979	9,804	9.91	0.841	1.80	416/306	189/314	152/116	60/90	19/21	14/10
38	18	2	1,904	4,469	4.69	0.955	2.13	575/229	39/26	145/24	49/10	17/9	5/11	83	54	2	915	4,027	8.80	0.948	1.79	164/115	106/291	67/36	25/51	11/28	8/12
39	54	7	1,780	5,460	6.13	0.871	1.97	443/236	144/129	159/126	68/74	7/5	28/19	84	116	12	2,531	9,667	7.64	0.866	1.86	554/413	204/232	189/174	89/85	22/17	29/24
40	60	7	1,867	5,686	6.09	0.903	1.94	415/212	241/227	174/78	96/89	32/24	13/7	85	34	5	1,749	6,155	7.04	0.939	1.92	424/237	137/138	129/54	64/65	19/21	12/15
41	73	13	2,045	4,463	4.36	0.897	2.14	558/217	166/162	179/47	86/57	21/26	5/11	86	99	20	2,979	10,234	6.87	0.812	1.91	671/439	282/432	253/149	106/129	37/58	35/23
42	74	3	1,757	8,417	9.58	0.902	1.78	346/322	150/449	134/257	59/162	16/49	26/12	87	18	5	1,871	2,559	2.74	0.968	2.60	619/147	58/35	117/29	62/13	10/3	12/6
43	127	1	*n.a.*	*n.a.*	*n.a.*	*n.a.*	*n.a.*	*n.a./n.a.*	*n.a./n.a.*	*n.a./n.a.*	*n.a./n.a.*	*n.a./n.a.*	*n.a./n.a.*	*88*	77	16	2,926	10,935	7.47	0.860	1.89	650/326	304/413	224/153	108/109	20/17	39/53
44	24	1	428	6,269	29.29	0.978	1.43	60/128	16/44	20/18	10/17	4/1	0/1	89	20	2	1,359	6,848	10.08	0.957	1.73	270/265	71/24	72/63	38/25	7/18	17/39
45	177	40	2,436	9,984	8.20	0.808	1.81	478/392	248/590	211/198	92/183	28/29	24/15	90	54	7	612	1,398	4.57	0.957	2.07	139/87	49/42	51/32	25/9	7/4	2/0

^A^Gene Interaction Layers 43,46,52,72 failed to construct; ^B^Modules counts are listed as MCL/LCM; ^C^Number of enriched terms in MCL/LCM modules.

To test the amount of unique function captured by k-means presorting, we compared the K=90 GIL set to networks constructed by randomizing the input samples at 50% and 100%, leaving cluster number and sizes intact. The total number of unique nodes (gene capture) decreased from 19,588 (at 0% randomization) to 13,568 (at 100% randomization), yet the number of unique edges increased from 558,023 (at 0%) to 705,399 (at 100%). Interestingly, this higher level of connectivity between smaller numbers of genes resulted in more MCL modules ranging from 39,931 (at 0%) to 47,623 (at 100%) as well as more total enriched function terms ranging from 37,614 (at 0%) to 64,236 (at 100%) (Additional file 1: Figure S2). However, many of these enriched functions were re-captured terms since the number of unique enriched terms was reduced from 4,789 (at 0%) to 3,518 (at 100%). Thus, K-means pre-sorting of datasets prior to network construction appears to have a positive effect on capture of unique biological function.

### Exploring GIL function

Gene expression samples in the K90 GIL collection were not segregated by specific experimental conditions, but rather segregated using knowledge-independent pre-clustering. Therefore, annotation of the experimental conditions of samples may help describe the biological context in each GIL. Experimental descriptions from the samples in GEO confer limited information but common keywords from these descriptions can provide valuable insight. Therefore, we attempted to annotate each GIL for biological context by using highly represented keywords from the GEO experiment descriptions. We expected two GILs would be more similar in function if they shared genes between modules. Therefore, GILs were ordered using average linked hierarchical clustering with shared node count between GIL LCMs as the similarity metric (Additional file 1: Figure S4). This procedure resulted in groups of GILs that showed non-random similarity in assigned keywords (*p* < 0.05) for keywords “leaf”, “root”, ”seed”, “seedling”, “iron”, and “mutant” (significant keywords are indicated as red boxes in Additional file 1: Figure S4). However, overlap in keywords is evident between GILs indicating that GILs are not solely comprised of samples from a single experimental condition.

**Figure 5:**
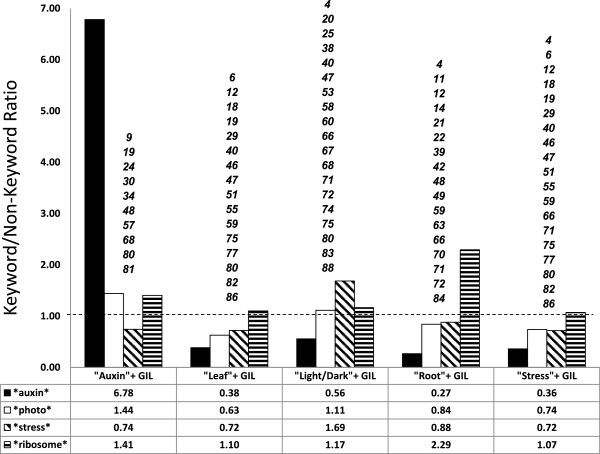
**Functional Term Enrichment Counting in Keyword Sorted GIL Sets.** The occurrence of keyword sub-patterns (auxin, photo, stress, ribosome) in LCM module enriched term descriptions was counted in GILs sorted by presence of high frequency keywords. Bars show the ratio of keywords within the GIL to GILs without the keyword. GIL identifier is shown in *italics*.

To determine if the GIL keyword assignment was biologically relevant, GILs were tested for an increase in relevant, enriched functional terms (Columns in Additional file 1: Figure S4). First, GILs were distributed into keyword and non-keyword groups for five different keywords (Figure 5). For example, 10 GILs with the “auxin” keyword were placed in an auxin group while the remaining 76 GILs were placed in a non-auxin group. Analogous groups were constructed for “leaf”, “light/dark”, “root” and “stress”. The number of specific enriched words from functional terms (e.g. *auxin*, *photo*, *stress*, *ribosome*; * = wildcard) found to be functionally enriched in LCMs were counted in both keyword and non-keyword groups. The ratio of these enriched terms between keyword and non-keyword groups can be found in Figure 5. For the auxin group, there was a 6.78 fold increase of enriched functional terms that contained “auxin”. In addition, we performed functional term enrichment on all genes in a GIL as final indicator of GIL context (Additional file 3: Table S2).

## Discussion

The partitioning method we describe dramatically increases the capture of significant co-expression relationships by segregating expression profiles into similar groups prior to correlation calculation. In effect, this approach is a dynamic significance thresholding strategy where a local hard threshold is determined for each GIL separately using the correlation matrix of each GIL. It is different from other dynamic thresholding methods which perform thresholding using the global correlation matrix. With pre-clustering of samples, correlation calculations occur locally for each GIL, allowing for existing threshold detection methods, such as RMT, to be used in a dynamic way. Naturally, there will be some false positives that surpass any thresholding method, but the lack of knowledge of true positive co-expression relationships make sensitivity and specificity difficult to quantify.

A second benefit for pre-clustering is reduction of complexity that results from mixing of samples from various conditions. It has been shown that as the number of conditions in a sample set increase, the distribution curve of correlation changes such that most correlations are centered around zero [26]. Thus, there are fewer high correlations and fewer significant co-expression relationships, and hence smaller networks as more conditions are present. Pre-sorting of samples groups them by similar overall expression levels, reduces the complexity of the dataset and mimics separation of samples by condition.

A third benefit to un-supervised pre-sorting of samples is that limited human understanding does not constrain the network. For example, grouping a set of samples into a control group (condition #1) and a disease state group (condition #2), may overlook changes in gene expression due to differences in genotype, tissue type and environmental conditions between the various individuals in the study. Even for carefully designed studies, confounding effects due to unrecognized or immeasurable conditions may hide subtle expression relationships. By pre-sorting without bias towards specific conditions, the underlying expression levels of each sample dictate membership in a group. The resulting GIL can therefore be multi-conditional which is more realistic than the idea that co-expression within a GIL is specific to a single condition. As mentioned previously, we do not see a single condition present for a GIL, although we did see some evidence that some GILs may be more representative of certain biological contexts.

Fourth, the GIL collection provides a novel framework for exploration of gene co-expression. We do not combine the co-expression relationships from all GILs into a single global network--GILs remain as separate entities. The modules within a single GIL provide a unique set of relationships for a unique biological function. A module in one GIL with a similar set of genes as a module in another GIL may have different co-expression relationships. This redundancy provides a realistic framework where genes and pathways are represented in different condition contexts. While the exact biological context of each GIL is unknown, differential connectivity between similar modules can indicate how co-expression changes between genes of interest. It is clear from our attempts to annotate the GILs that more research is required to assign conditions to the GILs and place genes and gene interactions into experimental contexts.

A disadvantage to K-means clustering for pre-sorting of input samples is that samples can only belong to a single GIL. It seems realistic to believe that the gene expression in one sample could share similarity with samples from several GILs. Biclustering draws on this idea that gene co-expression is a product of overlapping genes and samples. Also, Ruan *et. al.* constructed a sample co-expression network where samples were nodes and edges existed when samples shared similar expression patterns [52]. They show that module detection of a “sample network” yields modules with samples that share a similar biological context (e.g. lymphoma cell types). It seems logical to therefore use a link-community algorithm for detecting sample modules in such a sample network, from which we could therefore construct GILs where samples can be members of multiple GILs. Additionally, we used K-means clustering to segregate the expression datasets, but other clustering approaches, such as that proposed by Ruan et. al, or other commonly used clustering techniques, could also be effective in pre-sorting of samples.

Finally, is the *A. thaliana* interactome represented by the *K*90 GIL compendium comprehensive? While we detected 558,022 unique gene interactions, there were 1,584,378 total interactions, as well as gene modules that intersect through shared nodes between GILs. While an accurate protein coding gene count for *A. thaliana* has yet to be determined, a recent tally identified 27,416 loci and 35,386 transcript variants (TAIR10 build). The ATH1 microarray platform interrogates 20,677 of these known genes albeit with little transcript variant discriminatory power. The *K*90 GIL collection captured 19,588 loci in 558,022 temporal gene interactions. Extrapolating to include genes not measured by the array, we estimate the unique Arabidopsis co-expression interactome to be a minimum of 589,045 binary interactions. While this number is certain to increase with higher resolution RNA expression measurements (e.g. deep RNAseq sampling) obtained under additional experimental conditions, it does provide a baseline model of the RNA co-expression interactome.

An interactome estimate on a 10^5^ scale is not unprecedented for *A. thaliana*. A recent *A. thaliana* study estimated the baseline protein interaction space to be 299,000 ± 79,000 (μ ± SD) without accounting for protein isoforms. This estimate was based on approximately 2% (2,661 peptides) of the predicted interactome [53]. While not statistically significant, 2.1% (237/11,374 edges) of the PPI interactions overlapped with the *K*90 GIL collection. It is intriguing that a PPI network predicted to represent 2% of the interactome overlaps with 2% of interactome modeled by the GIL collection. We hypothesize that the capture of interactome space represented in the *K*90 GIL collection approaches the maximal interaction space of the measured genes across all the input samples.

## Conclusions

Our results indicate that pre-clustering transcriptome measurements by expression similarity prior to co-expression network construction captures more significant co-expression relationships than networks constructed without pre-clustering. In summary, we believe that this approach is simple, reduced-bias, and practical to reduce noise in large expression dataset collections. The enhanced resolution afforded by the GIL collection provides a more holistic platform for improved identification of gene modules that can be interrogated for novel biochemical pathways, used to assign new genes to known pathways, predict gene sets causal for important complex traits, and indicate pleiotropic relationships within and between modules derived from specific GILs in the GIL collection. The *A. thaliana K*90 GIL compendium described here is therefore the most comprehensive and we believe the most natural model of the gene co-expression interactome for a plant species. The method should be applicable to any organism where a large number of gene expression profiling datasets have been generated.

## Methods

### Partitioning expression datasets by K-means clustering

Expression measurements used for network construction were obtained from publicly available Affymetrix *Arabidopsis* ATH1 Genome Array experiments available in NCBI GEO (platform GPL198). A total of 7,158 samples were obtained in November 2011. RMA normalization [42] was performed for all samples together using the command-line utility of RMAExpress (http://rmaexpress.bmbolstad.com). Sample outlier detection was performed using the arrayQualityMetrics [54] tool for Bioconductor [55]. Samples that failed two of the three outlier tests were removed from the dataset. The normalized expression file, an *n* × *m* matrix of expression values with 22,746 rows of probe sets (no control) and 7,105 columns of samples (Additional file 2: Table S1), was clustered by similarity of expression values using *K*-means clustering via the *kmeans* function of R. *K*-means clustering was repeated for different values of *K* at 30, 60, 70, 80, 90, 120, 150, 180, and 210.

### Co-expression network construction

Each cluster of samples derived from the *K*-means clustering was used to construct individual networks—one for each cluster. Additionally, a “global” network was constructed consisting of all available samples. The network construction process was performed on each dataset cluster which included: 1) RMA normalization of the clustered datasets and not all possible arrays; 2) removal of control probes; 3) outlier sample removal; 4) removal of ambiguous probe sets; 6) calculation of a similarity matrix of pair-wise Pearson correlation values between all probe sets; 7) use of Random Matrix Modeling (RMT) [9] to identify a significant threshold to cull the similarity matrix; 8) conversion of the thresholded ATH1 probeset similarity matrix to an *Arabidopsis* gene network; and 9) module detection. Details specific to each step are further described.

The software package RMAExpress (http://rmaexpress.bmbolstad.com) was used to normalize each cluster individually. The samples of each cluster were provided as input, and the Chip Description File (CDF) was obtained from the Affymetrix website. A file containing an *n* x *m* expression matrix of normalized expression values, where *n* is the number of probe sets and *m* is the number of samples, was generated for each cluster. The rows of the expression matrix were then reduced in size through the removal of control probes using an in-house Perl script. Outlier samples within the cluster itself were identified using the arrayQualityMetrics [54] tool for Bioconductor [55]. The columns of the expression matrix were then reduced in size through removal of the outlier samples using an in-house Perl script. The rows of the expression matrix were then reduced again to remove any ambiguous probesets. Ambiguous probe sets are those that potentially hybridize to multiple genes in the *Arabidopsis* genome. Ambiguous probe sets were identified using megablast from the NCBI BLAST [56] package to align a FASTA file of probe sequences obtained from the Affymetrix website with the Arabidopsis coding sequences (CDS) obtained from the TAIR website (Lamesch et. al. 2012). Parameters for megablast set the word size to 25 (-W 25, the length of the probe sequence), and disabled low-complexity filtering (-F F). An in-house Perl script counted the probe to gene mappings and identified ambiguous probe sets. Pearson correlation coefficients were calculated using optimized C-code and an *m* x *m* similarity matrix was produced, where *m* is the number of remaining probe sets. Random Matrix Theory, which employs threshold detection using the nearest neighbor spacing distribution of eigenvalues, was used to identify an appropriate threshold for filtering the similarity matrix [9]. Optimized C-code, produced in-house, was used for RMT thresholding. An adjacency matrix is effectively produced by ignoring values (or setting values to zero) in the similarity matrix that are less than the threshold. Using an in-house Perl script, a network file was constructed, and edges in the network correspond to probe sets in the adjacency matrix that have a non-zero correlation value. Using mappings of probe set to TAIR gene models derived earlier with megablast, the probe set-based network was converted to a gene-based network. Edge lists for all K90 GILs and the Global network can be found in Additional file 4 and online at sysbio.genome.clemson.edu. Finally, modules, or sets of closely linked genes, were identified in each of the networks using the link community method [51] which circumscribes edges into communities using the ‘linkcomm’ binary version 1.0-4 in R [57] or MCL modules using the ‘mcl’ binary version 11-294 (http://micans.org/mcl/). Gene assignment to modules can be found in Additional file 5: Table S3.

### Functional enrichment analysis

Functional enrichment was performed for each of the modules from each GIL. Functional terms used for enrichment include Gene Ontology (GO) [46], InterPro [58], AraCyC [50], Plant Ontology (PO) [49], and KEGG [47] terms. Mappings of GO, PO, AraCyC, and InterPro terms to genes were obtained from the TAIR website (Lamesch et. al. 2012). KEGG terms were obtained by uploading Arabidopsis coding sequences (CDS) to the KEGG/KAAS server which maps KEGG terms using a homology-based method [59]. Functional enrichment was then performed using an in-house Perl script that functions similar to the online DAVID tool [60,61]. The tool uses a Fisher’s Exact test to look for significant over-representation of terms within a module versus the TAIR10 genes represented on the ATH1 platform as background. Module functional enrichment results (Bonferroni adjusted p < 0.001) can be found in Additional file 6: Table S4. Network functional enrichment results (Bonferroni adjusted p < 0.001) can be found in Additional file 3: Table S2.

### Network topology analysis

Network topology parameters were determined using the Netstat, Centrality, and Kronfit ‘example applications’ implementing the Stanford Network Analysis Project (SNAP) library Ver. 2011-12-31 (http://snap.stanford.edu/snap). The scaling exponent (γ) was estimated using the ‘power.law.fit’ function in the *igraph* R package version 0.5.4 (http://igraph.sourceforge.net/) under 64-bit R version 2.10.1.

### Semantic analysis

First, we counted all words in each GEO experiment description. After filtering for uninformative words from a custom dictionary, we listed the top ten words found in each GIL collection. These words were then manually inspected for RNA source (e.g. “anther” “leaf”), treatment (e.g. “light” ”dark”), “stress”, and “mutant”. These frequent keywords were then assigned to the respective GIL (Additional file 1: Figure S4; Additional file 7: Table S5).

## Competing interests

The authors declare no competing interests.

## Supplementary Material

Additional file 1: Figure S1 Additional topological metrics of the GIL constructed from varied partitioning sizes. **Figure S2.** Functional enrichment in MCL modules. **Figure S3.** Module discovery in GIL collections. **Figure S4.** Keyword assignment and shared node sorting of the K90 GIL collection. Click here for file

Additional file 2: Table S1 GEO Experiment Assignments to K90 GILs. Click here for file

Additional file 3: Table S2 Functional Enrichment Results for Global, K90 GIL Networks. Click here for file

Additional file 4 **Coexpression networks.** From-To Edge Lists for Global, K90 GILs with Correlation Values. Click here for file

Additional file 5: Table S3 Gene Assignments to Global, K90 GIL Modules. Click here for file

Additional file 6: Table S4 Functional Enrichment Results for Global, K90 GIL Modules. Click here for file

Additional file 7: Table S5 Filtered Keywords from GEO Experiment Descriptions Used in GIL Annotation. Click here for file
