# Supplementary material for: Maximizing capture of gene co-expression relationships through pre-clustering of input expression samples: an Arabidopsis case study
Source: BMC Syst Biol. 2013 Jun 5;7:44. doi: 10.1186/1752-0509-7-44 (PMC3679940; doi:10.1186/1752-0509-7-44)
Supplement: Additional file 1: Figure S1 — Additional topological metrics of the GIL constructed from varied partitioning sizes. Figure S2. Functional enrichment in MCL modules. Figure S3. Module discovery in GIL collections. Figure S4. Keyword assignment and shared node sorting of the K90 GIL collection. [file 1752-0509-7-44-S1.ppt]

## Slide 1
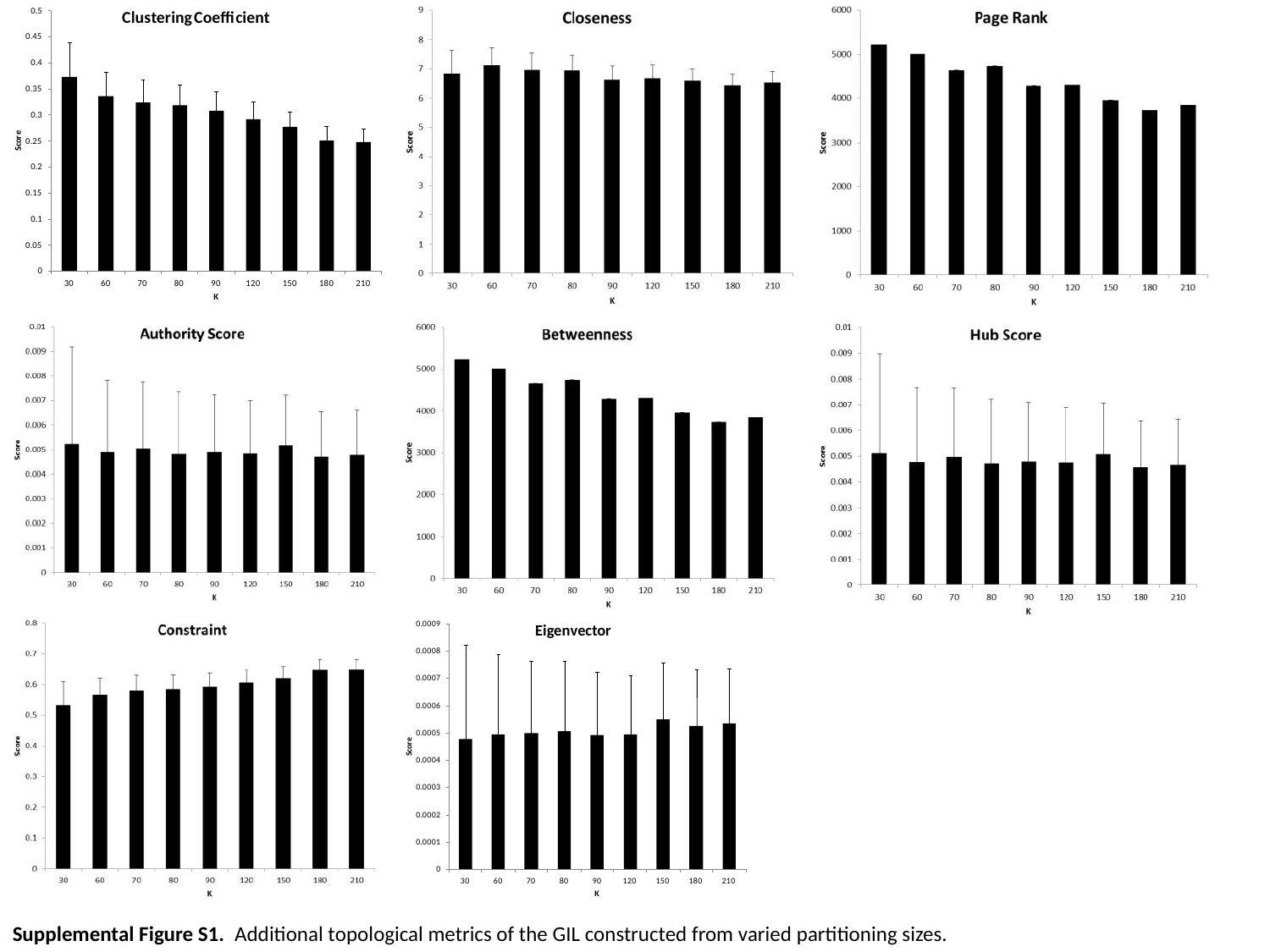

Supplemental Figure S1. Additional topological metrics of the GIL constructed from varied partitioning sizes.

## Slide 2
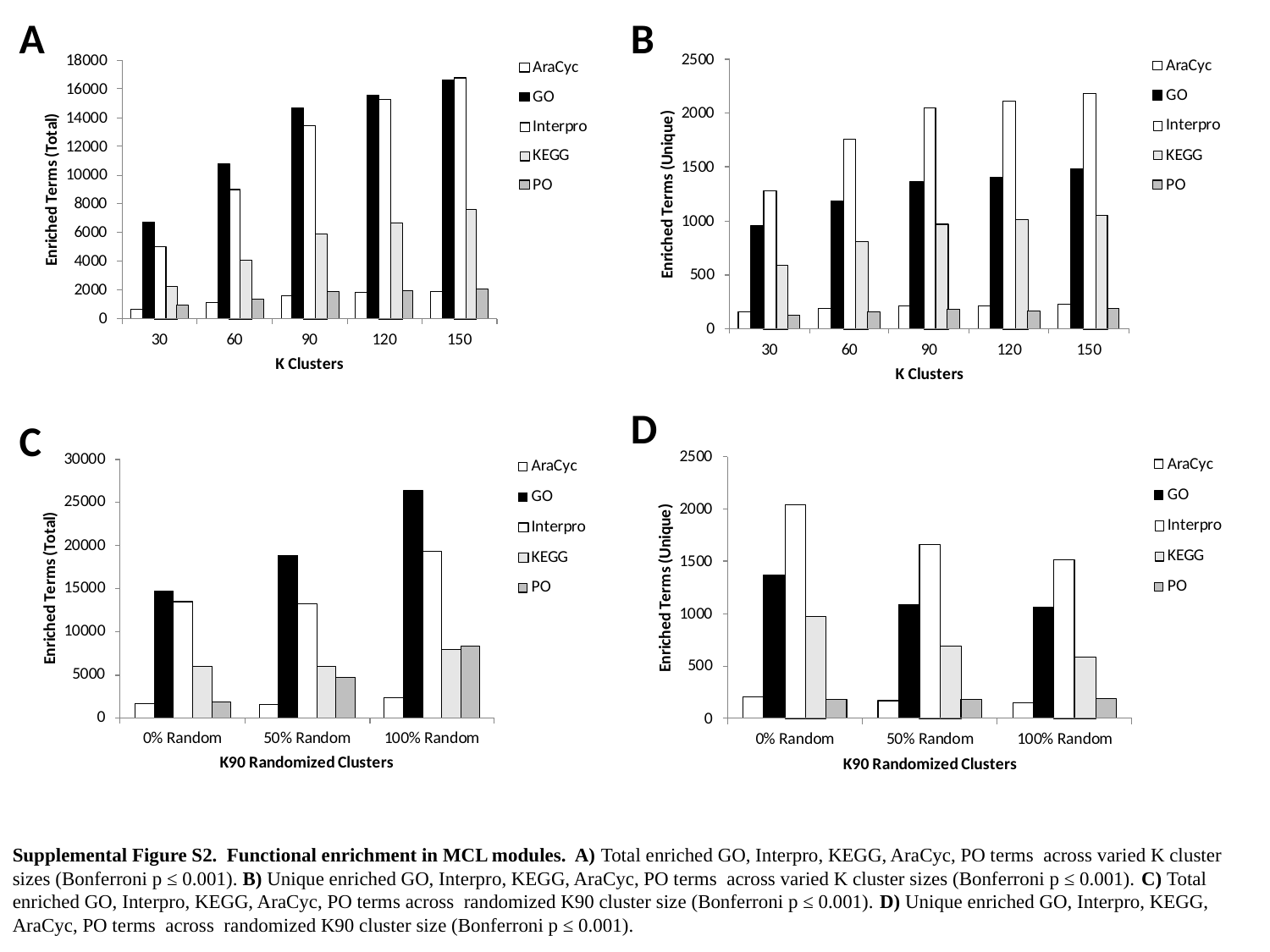

A
B
D
C
Supplemental Figure S2. Functional enrichment in MCL modules. A) Total enriched GO, Interpro, KEGG, AraCyc, PO terms across varied K cluster sizes (Bonferroni p ≤ 0.001). B) Unique enriched GO, Interpro, KEGG, AraCyc, PO terms across varied K cluster sizes (Bonferroni p ≤ 0.001). C) Total enriched GO, Interpro, KEGG, AraCyc, PO terms across randomized K90 cluster size (Bonferroni p ≤ 0.001). D) Unique enriched GO, Interpro, KEGG, AraCyc, PO terms across randomized K90 cluster size (Bonferroni p ≤ 0.001).

## Slide 3
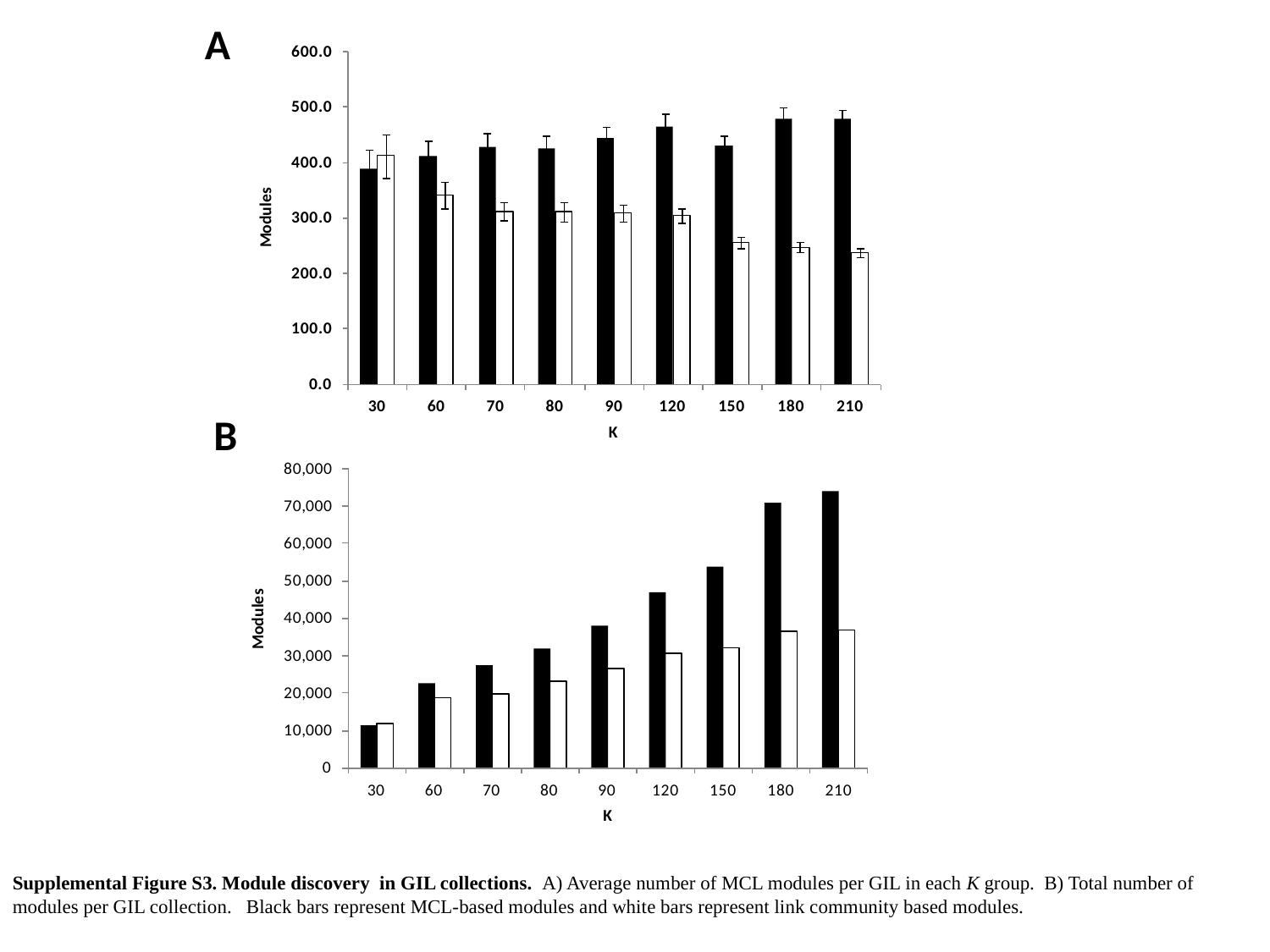

A
B
Supplemental Figure S3. Module discovery in GIL collections. A) Average number of MCL modules per GIL in each K group. B) Total number of modules per GIL collection. Black bars represent MCL-based modules and white bars represent link community based modules.

## Slide 4
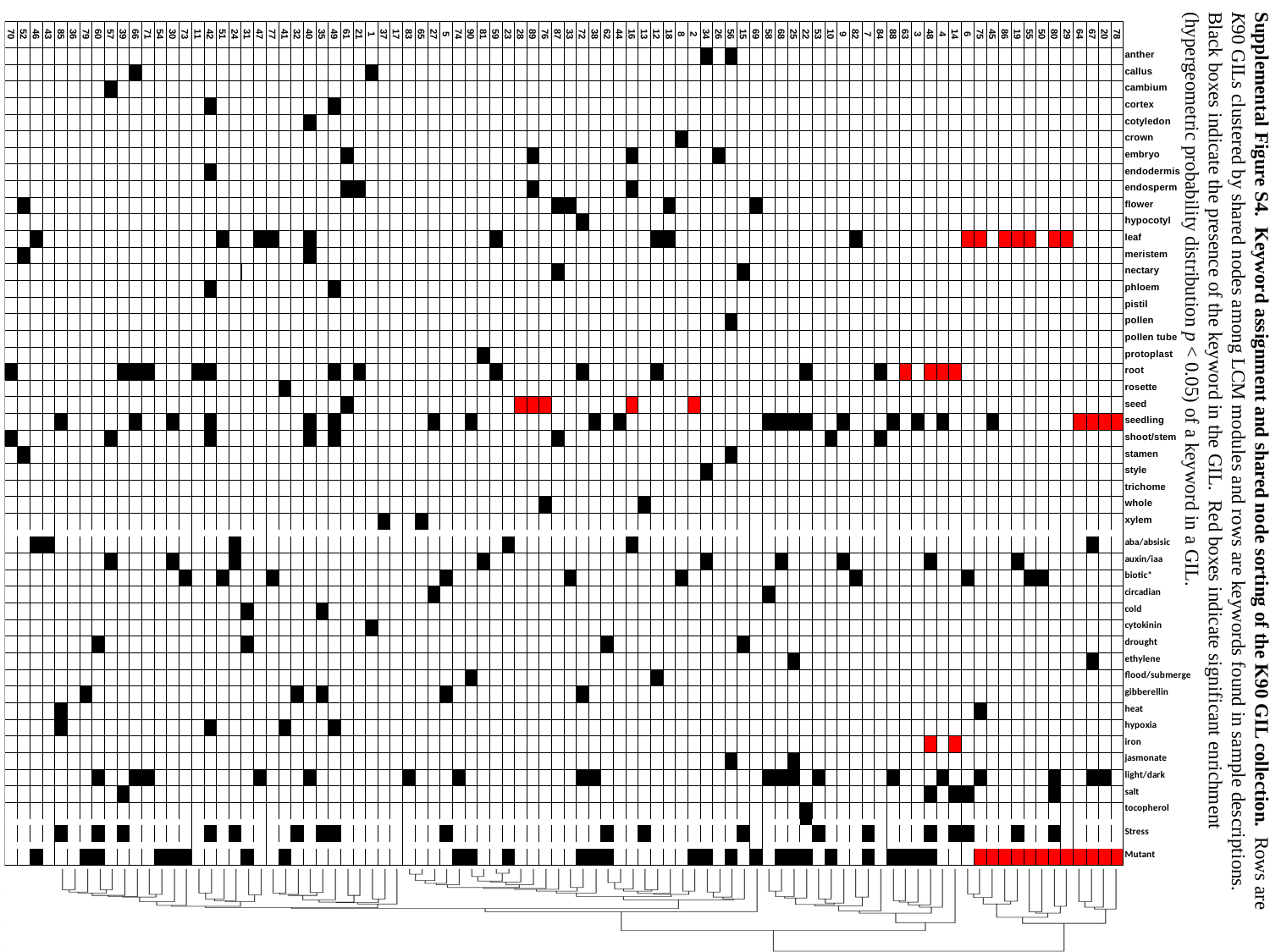

Supplemental Figure S4. Keyword assignment and shared node sorting of the K90 GIL collection. Rows are K90 GILs clustered by shared nodes among LCM modules and rows are keywords found in sample descriptions. Black boxes indicate the presence of the keyword in the GIL. Red boxes indicate significant enrichment (hypergeometric probability distribution p < 0.05) of a keyword in a GIL.
